# High-dose versus low-dose block-and-replace treatment for a first episode of Graves’ disease

**DOI:** 10.1530/ETJ-25-0039

**Published:** 2025-04-14

**Authors:** Arnaud Smolders, Aglaia Kyrilli, Stefan Matei Constantinescu, Bernard Corvilain, Chantal Daumerie, Maria-Cristina Burlacu

**Affiliations:** ^1^ Department of Endocrinology and Nutrition, Cliniques Universitaires Saint-Luc, Université Catholique de Louvain, Brussels, Belgium; ^2^ Department of Endocrinology, Hopital Erasme, Université Libre de Bruxelles, Brussels, Belgium

**Keywords:** antithyroid drugs, dose, Graves’ disease, block-and-replace

## Abstract

**Objective:**

The optimal treatment with antithyroid drugs (ATDs) for a first episode of Graves’ disease (GD) remains controversial.

**Methods:**

Retrospective, two academic centres study of newly diagnosed GD between 1990 and 2022, treated with ATD in block-and-replace (B+R) regimen for at least 12 months and followed up for at least 1 year after ATD discontinuation or until disease relapse. Sixty patients received high-dose B+R (HD) with fixed ATD dose maintained during the study, and 60 patients received low-dose B+R (LD) with lower ATD dose adjusted during the study.

**Results:**

Baseline characteristics were similar in both groups. The point-prevalence of euthyroidism was not different between HD and LD (38 vs 47%, *P* = 0.460 at 6 months, 69 vs 82%, *P* = 0.194 at 12 months, 70 vs 78%, *P* = 0.370 at 18 months, respectively). At 18 months, 27% HD vs 38% LD (*P* = 0.242) had thyroid eye disease. There were no differences in the number or type of ATD-related adverse events (AE) (no AE 73 vs 78%, *P* = 0.707). LD received mean lower ATD dose (15.3 ± 4.2 vs 30.0 ± 0.0 mg/day, *P* < 0.001) and lower levothyroxine dose (72.6 ± 16.7 vs 100.6 ± 24.5 μg/day, *P* < 0.001). After a first course of ATD, 63% of HD patients and 60% of LD patients relapsed (*P* = 0.707) after a median time (interquartile range) of 11.0 (18) vs 7.0 (19) months (*P* = 0.109).

**Conclusion:**

We observed similar relapse rates in patients with a first episode of GD receiving up to 50% less ATD and 30% less levothyroxine dose than high-dose B+R regimen.

## Introduction

Antithyroid drugs (ATDs) (propylthiouracil, methimazole and carbimazole, a pro-drug of methimazole) have become the first-choice treatment for newly diagnosed Graves’ disease (GD) worldwide (1, 2). ATD can be administered as monotherapy in a titration (T) regimen or in a block-and-replace (B+R) regimen, in which a replacement dose of levothyroxine (LT4) is added to a high, unchanged dose of ATD. First described in 1973 by Wise *et al.* (3) as a strategy to further lower TSH receptor antibodies by reducing the TSH stimulating effect, B+R treatment was credited in the 90s a higher remission rate of GD, but a subsequent meta-analysis of 26 randomised trials involving 3,388 participants showed no significant difference in relapse rate compared to T regimen (OR: 1.15, 95% CI: 0.79–1.67) (4). Contrary to the premise of Wise’ study, current evidence does not support a more stable thyroid function in patients under B+R compared to patients under T, as showed by one large retrospective (5) and a recent prospective multicentre study (6). These data, the assumption that taking less tablets will increase the compliance and the fact that B+R regimen was associated with more adverse effects than T regimen (4, 7) could favour the latter as the first-line medical treatment in GD (8, 9). However, several studies showed lower occurrence or little progression of thyroid eye disease (TED) in patients under B+R regimen (6, 10). During the rare studies using lower dose B+R regimen (10, 11), most of the patients maintained euthyroidism, even for prolonged periods. Depending on the presentation of GD, both B+R and T regimens could therefore be indicated and definitive conclusion about the optimal ATD treatment is still open to discussion.

This study analysed the effect of two different doses of B+R regimen on the evolution, complications and relapse rate of a first episode of GD treated with ATD.

## Materials and methods

**Figure 1:**
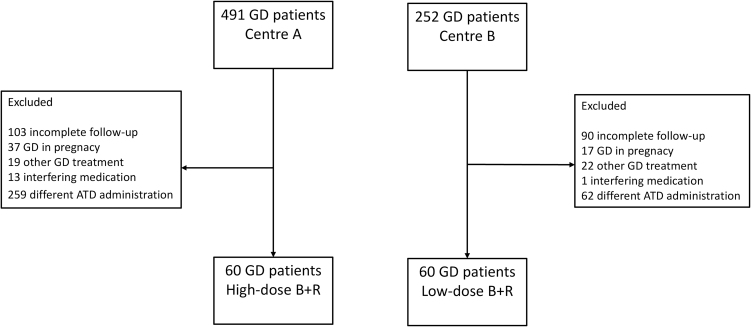
Flowchart of study’s patients.

This was a retrospective study conducted in two academic centres Hospital Erasme (centre A) and Cliniques Universitaires Saint-Luc (centre B) in Brussels, Belgium, in patients with newly diagnosed GD treated with ATD in a B+R regimen for at least 12 months and followed up for at least 1 year after ATD discontinuation or until disease relapse. From the initial 743 GD patients, we excluded 193/743 (26%) patients for incomplete follow-up, 41/743 (5.5%) for first-line treatments other than ATD for GD (e.g.: radioiodine or surgery), 54/743 (7%) for diagnosis of GD during pregnancy, 14/743 (2%) for medications interfering with thyroid function and 321/743 (43%) for different ATD administration, meaning administration of ATD in a regimen different from the one that was pre-defined as an inclusion criteria for each centre (HD for centre A and LD for centre B). The centre A used to treat GD patients with both HD and LD regimens, but the HD regimen was used mostly in the 90s and early 2000s. The patients excluded from centre A received either LD or titration. The centre B used exclusively LD as B+R regimen and the patients excluded from this centre received titration regimen. The assignment of patients to HD, LD or titration was not related to the GD presentation, but to the choice of the care giver. ATD treatment was stopped before 12 months in 16 patients from centre A and five patients from centre B due to the occurrence of ATD-related adverse events (AEs). We included the first 60 consecutive patients treated with high-dose B+R (HD) at centre A between 1990 and 2022 and 60 consecutive patients treated with low-dose B+R (LD) at centre B between 2010 and 2020. Figure 1 illustrates the study selection process in detail.

The diagnosis of GD was made on the basis of the association of low TSH, elevated free FT4 and/or elevated FT3, imaging compatible with GD and/or the presence of TRAb. The presence of TED was defined as one or more of the following eye findings: soft tissue changes (moderate or severe eyelid/conjunctival redness and moderate or severe eyelid/periorbital swelling), proptosis above the upper normal limit (Asians: 18 mm, Caucasians: 20 mm, African heritage: 22 mm), diplopia (intermittent, inconstant or constant) and decreased visual acuity attributable to TED. Relapse was defined as recurrent biochemical hyperthyroidism with the elevation of serum TRAb and/or suggestive imaging, or the necessity to perform total thyroidectomy after a first uninterrupted course of treatment.

### Block-and-replace regimens

Patients in HD group received a fixed dose of 30 mg per day methimazole (or equivalent; e.g. 100 mg PTU = 10 mg MMI) during the whole duration of the treatment, associated with a replacement dose of levothyroxine (LT4) adjusted to maintain thyroid hormones levels within the normal range. Patients in the LD group received 20–40 mg per day methimazole (or equivalent) during the first 1–3 months of treatment depending on the severity of hyperthyroidism, until thyroid hormones normalisation, then 10–20 mg per day, in association with an adjusted dose of LT4, aiming to keep thyroid hormones levels within the normal range.

### Thyroid function tests

Measurement of thyroid function parameters and thyroid antibodies were performed at the laboratory of each centre, with different assays with different reference values over time. The results were normalised by dividing obtained results by the upper normal limit of that assay, and then multiplied by the upper limit of the reference range used in centre B after June 2014 (according to the methodology described by Zarkovic *et al.*) (6).

Euthyroidism was defined as TSH and FT4 within reference ranges, hypothyroidism as TSH above upper normal range (with normal FT4 and FT3 levels in subclinical hypothyroidism) and hyperthyroidism as TSH below lower normal range (with normal FT4 and FT3 levels in subclinical hyperthyroidism).

The study was approved by the local Ethics Committees (Comité d’Ethique Hospitalo-Facultaire des Cliniques Universitaires St-Luc, Université Catholique de Louvain and Comité d’Ethique Erasme-ULB). Written informed consent was not required due to the retrospective nature of the study.

### Statistical analysis

Statistical analyses were performed using the IPSS Statistics© software from IBM© (version 28.0). A *P* value of less than 0.05 was considered significant. Continuous variables were described either as the mean ± standard deviation or median with interquartile range (IQR). Boxplots, histograms and density/Q-Q plots were used to assess the normality of the data. The Kolmogorov–Smirnov’s test was not used to describe normality given the size of the population. Discrete variables were described using their frequency. Subgroup analyses were performed using Pearson’s X^2^ test, Fisher’s test or Fisher–Freeman–Halton’s exact test for categorical unpaired variables. Student’s *t* test was used for comparing the means of continuous unpaired variables whereas Mann–Whitney’s test was chosen for comparing the medians. A general linear model was used to analyse the effect of time and treatment regimen on biological parameters (TSH, FT4, TRAb, ATD and LT4). We used the GraphPad Prism version 10.2.2 for illustrations.

## Results

### GD presentation and evolution

Baseline characteristics were similar in both groups (Table 1). Most patients were non-smoking Caucasian women and the severity and the duration of hyperthyroidism and TRAb levels at GD diagnosis were not different between HD and LD group.

**Table 1:** Baseline patients’ characteristics. Results shown as *n* (%), mean ± SD or median (IQR).

	HD	LD	*P*-value
Total *n*	60	60	
Age (years)	40.8 ± 13.1	44.9 ± 14.1	0.113
Females	45 (75%)	48 (80%)	0.512
Ethnicity			0.063
Caucasian	77%	75%	
Nord-African	18%	13%	
Sub-Saharian	2%	12%	
Asian	3%	0%	
Smoking status			0.151
Never	83%	70%	
Ex	7%	7%	
Current	10%	23%	
Symptoms duration before diagnosis (months)	4.2 ± 4.5	4.2 ± 3.4	0.990
TED: present	12 (20%)	13 (22%)	0.500
Goitre: present	39/58 (65%)	40/60 (67%)	0.947
FT4 (pmol/L)	46.3 ± 32.0	47.7 ± 26.9	0.810
TRAb (UI/L)	6.3 (8.4)	5.5 (9.7)	0.236

HD, high-dose block-and-replace; LD, low-dose block-and-replace; TED, thyroid eye disease; FT4, free T4; TRAb, TSH receptor antibodies.

After a first course of ATD, 63% HD patients and 60% LD patients relapsed (*P* = 0.707) after a median time (IQR) of 11.0 (18) vs 7.0 (19) months (*P* = 0.109) (Table 2). There were no differences regarding the relapses and time to relapse during the first year after treatment cessation. LD patients received the first ATD course for a longer time (20.9 ± 7.1 vs 17.5 ± 5.3 months, *P* = 0.003) and their TRAb levels at the end of ATD treatment were lower than in the HD group (0.3 (0.8) vs 1.44 (1.2) UI/L, *P* < 0.001). When we compared the 44 HD patients treated with ATD for only 12–18 months to the 28 LD patients treated for the same period of time, we observed no differences in relapse rate (66 vs 61%, *P* = 0.655) nor in the time to relapse (13 (18) vs 11 (20) months, *P* = 0.767). In addition, in these patients, TRAb levels at the end of ATD treatment were lower in LD patients (0.3 (0.7) vs 1.3 (1.2) UI/L, *P* < 0.001) (Table 2). The proportion of patients in whom TRAb were normalised at the end of the treatment was higher in LD group (86 vs 65%, *P* < 0.001) (Table 2). The total duration of follow-up was longer in the HD group (*P* = 0.008).

**Table 2:** GD evolution and relapse. Results shown as *n* (%), mean ± SD or median (IQR).

	HD		LD		*P* value
	*n*	Values	*n*	Values	
Treatment duration (months)	60	17.5 ± 5.3	60	20.9 ± 7.1	0.003
Relapse	60	38 (63%)	60	36 (60%)	0.707
Time to relapse (months)	60	11.0 (18)	60	7.0 (19)	0.109
Relapse first year	60	20 (33%)	60	24 (40%)	0.449
Patients treated 12–18 months with ATD					
Relapse	44	29 (66%)	28	17 (61%)	0.655
Time to relapse (months)	44	13 (18)	28	11 [20]	0.767
Follow-up duration (months)	60	66 (90)	60	28 (45)	0.008
End of ATD treatment					
TED	60	16 (27%)	60	23 (38%)	0.242
TRAb (UI/L)	60	1.4 (1.2)	60	0.3 (0.8)	<0.001
TRAb normalised	57	37 (65%)	58	50 (86%)	<0.001
TRAb after 12–18 months (UI/L)*	44	1.3 (1.2)	28	0.3 (0.7)	<0.001

HD, high-dose block-and-replace; LD, low-dose block-and-replace; ATD, antithyroid drug; TRAb, TSH receptor antibodies; TED, thyroid eye disease; GD, Graves’ disease.

* TRAb at the end of ATD treatment in patients treated 12–18 months with ATD

TED was present at diagnosis in one-fifth of patients in each group (*P* = 0.5) and 27% HD vs 38% LD (*P* = 0.242) had TED after 18 months of ATD treatment. Although not significantly different, there were always more new TED cases in the LD group (4 vs 2 at 6 months, 2 vs 1 at 12 months and 3 vs 1 at the end of treatment, p = ns for all). The prevalence of TED increased significantly in time in both groups (*P* < 0.001) (Tables 1 and 2).

### ATD treatment and AEs

Most patients of the two groups were treated with methimazole (MMI), but the ATD type was more often changed in HD patients (*P* = 0.01), although there were no differences in the number or type of drug-related AEs (no AE 73 vs 78%, *P* = 0.707) (Table 3). LD received significantly lower ATD dose (15.3 ± 4.2 vs 30.0 ± 0.0 mg/day, *P* < 0.001) and lower LT4 dose (72.6 ± 16.7 vs 100.6 ± 24.5 μg/day, *P* < 0.001) than HD (Table 3). ATD and LT4 doses were significantly lower in the LD group at each time point (6 months, 12 months and end of treatment) (*P* < 0.001 for all) (Supplementary Table 1 (see section on Supplementary materials given at the end of the article)).

**Table 3:** ATD cumulative dose and AEs during the ATD treatment. Results shown as *n* (%) or as mean ± SD.

	HD (*n* = 60)	LD (*n* = 60)	*P* value
ATD			0.01
MMI	80%	97%	
PTU	3%	0%	
Both	17%	3%	
ATD cumulative dose (mg/day)	30.0 ± 0.0	15.3 ± 4.2	<0.001
LT4 cumulative dose (μg/day)	100.6 ± 24.5	72.6 ± 16.7	<0.001
ATD AE			0.707
None	73%	78%	
1	22%	15%	
>1	5%	7%	
ATD liver toxicity	7 (12%)	4 (7%)	0.343
ATD haematological toxicity	3 (5.0%)	5 (8%)	0.717
ATD skin toxicity	7 (12%)	7 (11.67%)	1.000
ATD articular toxicity	1 (2%)	1 (2%)	1.000

HD, high-dose block-and-replace; LD, low-dose block-and-replace; ATD, antithyroid drugs; MMI, methimazole; PTU, propylthiouracil; LT4, levothyroxine; AE, adverse events.

### The evolution of the thyroid function during ATD treatment

**Figure 2:**
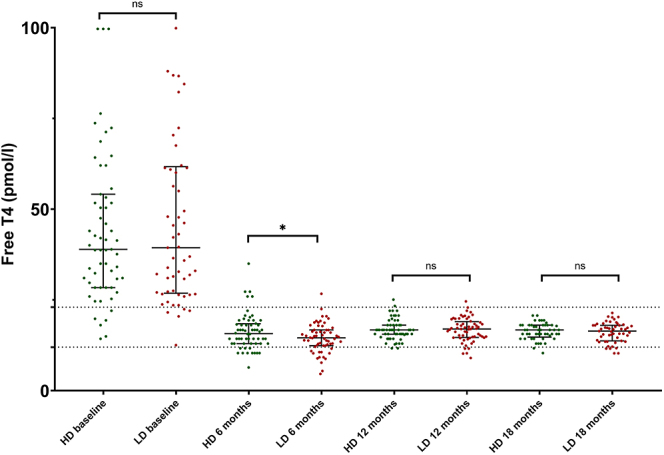
Evolution of free T4 levels in GD patients treated with a first course of high-dose (HD) or low-dose (LD) block-and-replace regimens. *P < 0.05; data shown as scatter plot with median (IQR).

**Figure 3:**
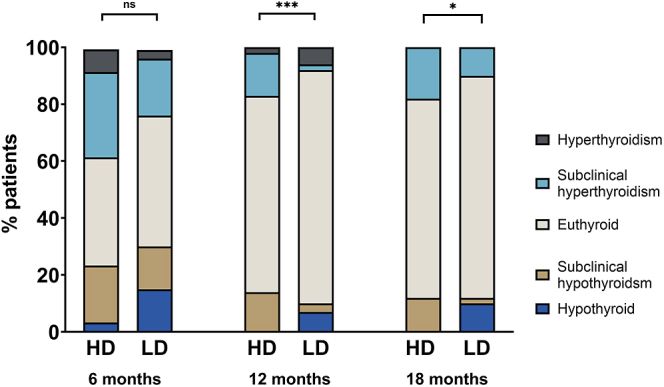
Thyroid function status during the treatment of GD with high-dose (HD) or low-dose (LD) block-and-replace regimens. *P < 0.05; ***P < 0.001.

Overall, there were no differences between groups regarding the evolution of the thyroid function during ATD (TSH *P* = 0.847, FT4 *P* = 0.895) (Table 4). The point-prevalence of euthyroidism was not different between HD and LD (38 vs 47%, *P* = 0.460 at 6 months, 69 vs 82%, *P* = 0.194 at 12 months, 70 vs 78%, *P* = 0.370 at 18 months, respectively), although LD had higher TSH (2.16 (4.0) vs 0.86 (2.9) mU/L, *P* = 0.206) and lower FT4 (14.5 ± 4.0 vs 16.2 ± 4.9 pmol/L, *P* = 0.037) than HD at 6 months (Table 4 and Fig. 2). However, significant differences appeared at 12 months and up to the end of the treatment regarding the prevalence of overt and subclinical hyper- or hypothyroidism (*P* = 0.10 at 6 months, *P* < 0.001 at 12 months and *P* = 0.015 at 18 months) (Fig. 3).

**Table 4:** The evolution of the thyroid function during ATD treatment. Results are shown as the mean ± SD or median (IQR).

	At diagnosis	6 months	12 months	18 months	*P* value	
					*P* (time)	*P* (treatment)
TSH (0.27–4.2 mU/L)					<0.01	0.847
High-dose B+R	0.04 (0.0)	0.86 (2.9)	1.72 (2.2)	1.26 (1.9)		
Low-dose B+R	0.01 (0.0)	2.16 (4.)	1.91 (1.6)	1.56 (1.5)		
FT4 (12–22 pmol/L)					<0.01	0.895
High-dose B+R	46.3 ± 32.0	16.2 ± 4.9	16.8 ± 2.8	16.3 ± 2.2		
Low-dose B+R	47.7 ± 26.9	14.5 ± 4.0	16.7 ± 3.2	16.0 ± 2.7		

B+R, block-and-replace; ATD, antithyroid drugs.

## Discussion

In this retrospective multicentre study, we observed similar relapse rates in patients with a first episode of GD treated for at least 1 year either with high-dose ATD or with a low-dose ATD B+R regimen. Our observations were made in a predominantly Caucasian GD population with similar characteristics in term of age at diagnosis, gender prevalence, smoking habits, severity of hyperthyroidism and TED prevalence as the general GD population (12). Our results reproduce the previous findings of a large, randomised European multicentre study of 309 GD patients treated with two fixed MMI dosages (10 vs 40 mg) with LT4 supplementation, showing similar relapse rate and time to relapse in the two groups (11). In relapsing GD patients, it was shown that there is no dose-related effect of MMI on the intensity of the intrathyroidal autoimmune process (13). As an example, although, by definition, the ATD dose used in B+R regimen is higher than in T regimen, neither regimen is superior in term of GD relapse (4). In addition, in a prospective study of 310 GD Japanese patients assigned to either MMI 15 mg + inorganic iodine/day or MMI 30 mg/day regimen, there was no difference in the remission rate between groups (14). These authors concluded that higher MMI doses were not justified for the GD treatment, not only because of no difference in relapse rate, but also because of higher prevalence of drug-related adverse effects. Indeed, in the study by Reinwein *et al.*, euthyroidism was achieved earlier in the higher MMI dose group at the expense of an increased rate of adverse reactions (26 vs 15.5%, *P* < 0.01) (11). In the study by Sato *et al.*, (14), the proportion of patients achieving normal FT4 level in ≤30 or ≤60 days was significantly smaller and the adverse effects that required discontinuation of MMI were more frequent in the MMI 30 mg-treated group (14.8 vs 7.5%, *P* = 0.0387). We, and Zarkovic *et al.* in a study comparing B+R vs T regimen (6), did not observe a difference in the number or type of ATD-related adverse effects with different ATD regimens. However, in our study, the ATD type was more often changed in high-dose B+R patients. This might be explained by a different management of ATD toxicities in the centre using high-dose regimen, consisting in a more frequent switch from MMI to PTU in case of mild adverse effects, instead of using symptomatic treatment. Moreover, there were more patients from centre A who were excluded for the occurrence of ATD-related AEs during the first 12 months of ATD treatment.

The proportion of euthyroid patients at 6 months in our study (38 and 47%) was much lower than the proportion of patients achieving early euthyroidism in the study by Reinwein *et al.* (11), where 84.9% of patients receiving 10 mg MMI and 91.6% of patients receiving 40 mg MMI were already euthyroid after 6 weeks of treatment. This difference might be explained by a different definition of euthyroidism (normal thyroid hormones irrespective of the TSH level in the study by Reinwein vs normal TSH and T4 levels in our study). In the study by Zarkovic *et al.* (6) that used a similar definition of euthyroidism as our study, the proportion of B+R patients who achieved euthyroidism at 6 months was only 24.5%. Moreover, different severity of the hyperthyroidism between studies could also have influenced the time to euthyroidism.

Current guidelines (1, 9) recommend the measurement of TSH receptor antibody (TRAb) levels before stopping ATD therapy, TRAb persistence being predictive of GD relapse. TRAb levels were lower and more often normalised in LD patients at the end of the first ATD course, but these patients have been treated for a longer period than HD patients. However, we observed the same difference in TRAb levels when only patients treated with ATD for 12–18 months were compared. Another factor that could have influenced the TRAb evolution is the variability of the thyroid function that was higher in the HD group. Both hyper- and hypothyroidism can favour the persistence of TRAbs (15) and during the ATD treatment there were, although not significantly, less euthyroid patients at each time-point in the HD group and more patients with both overt and subclinical hyper- or hypothyroidism after 1 year of treatment in this same group. We could not determinate if these variations of the thyroid function were related to differences in the frequency of blood tests controls, higher levothyroxine dose or worse compliance with ATD treatment in the HD group.

In a prospective multicentre study comparing B+R vs T regimen, the fluctuations of the thyroid function during the first year of treatment could have explained a higher occurrence of TED in the latter group (17.8 vs 9.1%, *P* = 0.096) (6). We also observed more TED cases by the end of ATD treatment in the LD group in whom, after 6 months of ATD, TSH was higher and FT4 lower than in the HD group, although the difference was not significant for the TSH. These observations could suggest that GD patients who present with more severe hyperthyroidism or with TED would benefit more from a higher ATD dose treatment. In our opinion, the risk of TED occurrence or progression related to the choice of ATD treatment should be part of shared decision-making in the management of GD.

Despite a longer duration of treatment, the LD strategy allowed to administer up to 50% less ATD and 30% less LT4 dose than HD regimen, with the same efficacy in term of disease remission. The cost-effectiveness of the dose reduction should be assessed by further studies, but have to be considered at a time when a persistent shift towards ATD therapy was noted in the management of GD worldwide (2).

Our study has several limitations related to its retrospective design. It was realised in two different centres that could have had different strategies about the patients’ follow-up and the management of drug-related adverse effects. We did not evaluate the frequency of blood tests that could have influenced the stability of the thyroid function. Moreover, our conclusion about the relapse rate could be challenged by the fact that there were more patients from centre B than centre A who received radio-iodine or surgery as a first-line treatment and by the much shorter follow-up of the LD group, which nevertheless exceeded 12 months, most of the GD relapses being observed during the first year after ATD cessation (1, 9). Our conclusions could concern only a population who, like ours (16), is mildly iodine-deficient, the MMI dose needed to control hyperthyroidism under this condition being lower (11).

## Conclusion

In conclusion, we observed similar relapse rates in patients with a first episode of GD receiving up to 50% less ATD and 30% less LT4 dose than in high-dose B+R regimen. However, we identified notable disparities in the incidence of thyroid dysfunction during treatment, with patients receiving high-dose demonstrating elevated levels TRAb and a reduced occurrence of normal TRAb levels. This, together with slight differences in TED occurrence during ATD treatment, adds to the debate about how to best individualise this therapy in each patient.

## Supplementary materials



## Declaration of interest

The authors declare that there is no conflict of interest that could be perceived as prejudicing the impartiality of the work reported.

## Funding

This work did not receive any specific grant from any funding agency in the public, commercial or not-for-profit sector.

## Author contribution statement

MCB wrote the first draft. AS collected the clinical data and AS and SMC performed all statistical analyses. MCB, AK, BC and CD cared for patients. MCB led the clinical study. All authors reviewed and approved the final manuscript.

## Data availability

The data that support the findings of this study are not publicly available due their containing information that could compromise the privacy of research participants but are available from MCB upon reasonable request.
