# Supplementary material for: High-dose versus low-dose block-and-replace treatment for a first episode of Graves’ disease
Source: Eur Thyroid J. 2025 Apr 14;14(2):e250039. doi: 10.1530/ETJ-25-0039 (PMC12020463; doi:10.1530/ETJ-25-0039)
Supplement: Supplementary file 1 [file supplementary_materials.pdf]

Supplemental Table 1. The differences between cumulative doses of ATD and LT4 administered in each centre at each time point.

	0-6 months	6-12 months	12 months-end of treatment	p
**ATD dose (mg/day)**				p(time) < 0.001
**High-dose B+R**	30.0 ± 0.0	30.0 ± 0.0	30.0 ± 0.0	p(treatment) < 0.001
**Low-dose B+R**	19.5 ± 4.5	14.9 ± 4.8	12.6 ± 5.0	
**LT4 dose (mcg/day)**				p(time) <0.001
**High-dose B+R**	87.6 ± 21.4	107.4 ± 29.8	111.0 ± 32.3	p(treatment) < 0.001
**Low-dose B+R**	63.5 ± 12.5	76.3 ± 20.2	79.1 ± 23.2	

Results shown as mean ± s.d. B+R: block-and-replace; ATD: antithyroid drugs; LT4: levothyroxine
